# Chromosome 15q24 microdeletion syndrome

**DOI:** 10.1186/1750-1172-7-2

**Published:** 2012-01-04

**Authors:** Pilar L Magoulas, Ayman W El-Hattab

**Affiliations:** 1 Department of Molecular and Human Genetics, Baylor College of Medicine, Houston, TX, USA; 2 Division of Medical Genetics, Department of Child Health, University of Missouri Health Care, Columbia, MO, USA

## Abstract

Chromosome 15q24 microdeletion syndrome is a recently described rare microdeletion syndrome that has been reported in 19 individuals. It is characterized by growth retardation, intellectual disability, and distinct facial features including long face with high anterior hairline, hypertelorism, epicanthal folds, downslanting palpebral fissures, sparse and broad medial eyebrows, broad and/or depressed nasal bridge, small mouth, long smooth philtrum, and full lower lip. Other common findings include skeletal and digital abnormalities, genital abnormalities in males, hypotonia, behavior problems, recurrent infections, and eye problems. Other less frequent findings include hearing loss, growth hormone deficiency, hernias, and obesity. Congenital malformations, while rare, can be severe and include structural brain anomalies, cardiovascular malformations, congenital diaphragmatic hernia, intestinal atresia, imperforate anus, and myelomeningocele. Karyotypes are typically normal, and the deletions were detected in these individuals by array comparative genomic hybridization (aCGH). The deletions range in size from 1.7-6.1 Mb and usually result from nonallelic homologous recombination (NAHR) between paralogous low-copy repeats (LCRs). The majority of 15q24 deletions have breakpoints that localize to one of five LCR clusters labeled LCR15q24A, -B, -C, -D, and -E. The smallest region of overlap (SRO) spans a 1.2 Mb region between LCR15q24B to LCR15q24C. There are several candidate genes within the SRO, including *CYP11A1, SEMA7A, CPLX3, ARID3B, STRA6, SIN3A *and *CSK*, that may predispose to many of the clinical features observed in individuals with 15q24 deletion syndrome. The deletion occurred as a *de novo *event in all of the individuals when parents were available for testing. Parental aCGH and/or FISH studies are recommended to provide accurate genetic counseling and guidance regarding prognosis, recurrence risk, and reproductive options. Management involves a multi-disciplinary approach to care with the primary care physician and clinical geneticist playing a crucial role in providing appropriate screening, surveillance, and care for individuals with this syndrome. At the time of diagnosis, individuals should receive baseline echocardiograms, audiologic, ophthalmologic, and developmental assessments. Growth and feeding should be closely monitored. Other specialists that may be involved in the care of individuals with 15q24 deletion syndrome include immunology, endocrine, orthopedics, neurology, and urology. Chromosome 15q24 microdeletion syndrome should be differentiated from other genetic syndromes, particularly velo-cardio-facial syndrome (22q11.2 deletion syndrome), Prader-Willi syndrome, and Noonan syndrome. These conditions share some phenotypic similarity to 15q24 deletion syndrome yet have characteristic features specific to each of them that allows the clinician to distinguish between them. Molecular genetic testing and/or aCGH will be able to diagnose these conditions in the majority of individuals.

**Disease name and synonyms:**

Chromosome 15q24 deletion syndrome

15q24 deletion syndrome

15q24 microdeletion syndrome

## Definition and diagnostic criteria

**Figure 1:**
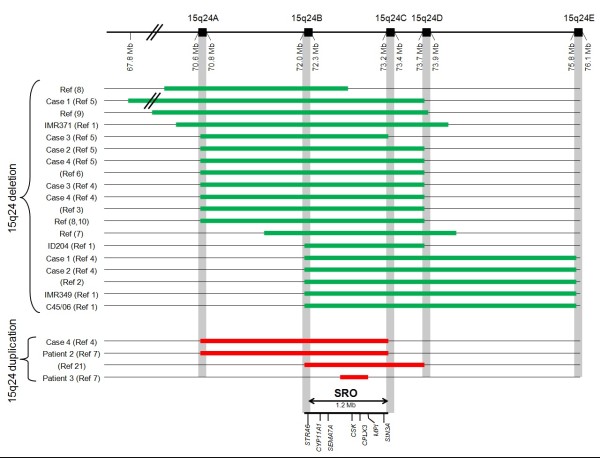
**Schematic diagram showing approximate size, position (NCBI36/hg18 assembly), gene, and breakpoint locations of reported cases of 15q24 microdeletions (upper section) and 15q24 microduplications (lower section)**. Reference and case number are located in the boxes to the left of the diagram. Genes within the SRO are indicated below the diagram.

Chromosome 15q24 microdeletion syndrome is a rare and novel microdeletion syndrome characterized by pre- and post-natal growth retardation, intellectual disability, distinct facial features, and genital, skeletal, and digital anomalies. Additional common features include hypotonia, behavioral problems, and recurrent infections (table 1) [1-10]. Although some of the characteristic features may be non-specific, the combination of these findings should prompt the clinician to suspect this diagnosis. Most of the deletions are between 1.7 to 6.1 megabases (Mb) in size, with the smallest region of overlap (SRO) spanning a 1.2 Mb region (Figure 1) [1-10]. Routine high-resolution karyotypes typically do not detect the deletion and all individuals with 15q24 deletion syndrome were diagnosed by using array comparative genomic hybridization (CGH) [1-10].

**Table 1:** Summary of clinical manifestation in patients with 15q24 microdeletion syndrome

*Characteristic*	*Cases*
Gender	Male - 16/19 (84%)

*De novo *occurrence	18/18 (100%)

**Developmental delay**	19/19 (100%)

**Growth abnormalities**	**10/18 (56%)**

Low birth weight/IUGR	6/18 (33%)

Short stature	5/18 (28%)

Obesity	3/18 (17%)

Microcephaly	3/18 (17%)

Feeding difficulties	4/18 (22%)

**Distinct Facial features**	**19/19 (100%)**

Long face	6/18 (33%)

Facial asymmetry	5/18 (28%)

High anterior hairline	12/18 (67%)

Epicanthal folds	9/18 (50%)

Hypertelorism	8/18 (44%)

Downslanting palpebral fissures	9/18 (50%)

Sparse, broad medial eyebrows	9/18 (50%)

Strabismus	7/18 (39%)

Nystagmus	2/18 (11%)

Broad nasal base	6/18 (33%)

Depressed nasal bridge	5/18 (28%)

High nasal bridge	2/18 (11%)

Ear abnormalities	11/18 (61%)

Palate abnormalities	4/18 (22%)

Long smooth philtrum	10/18 (56%)

Full lower lip	7/18 (39%)

Small mouth	6/18 (33%)

**Genital abnormalities**	**11/18 (61%)**

Hypospadias	6/15 (40%)

Microphallus	5/15 (33%)

Cryptorchidism	3/15 (20%)

**Digit abnormalities**	**16/18 (89%)**

Thumb abnormalities	7/18 (39%)

Brachydactyly/short digits	6/18 (33%)

Clinodactyly	3/18 (20%)

Toe abnormalities	6/18 (33%)

**Skeletal abnormalities**	**11/18 (61%)**

Joint laxity	10/18 (56%)

Scoliosis/kyphosis	6/18 (33%)

**Neurologic findings**	**15/19 (79%) **

Hypotonia	11/18 (61%)

Behavior problems	7/19 (37%)

MRI abnormalities	8/18 (44%)

**Other features**	

Recurrent infections	7/18 (39%)

Hernias	5/19 (26%)

Congenital heart disease	4/18 (22%)

Hearing loss	4/18 (22%)

Diaphragmatic hernia	2/18 (11%)

Intestinal atresia	2/18 (11%)

Imperforate anus	1/18 (6%)

Coloboma	1/18 (6%)

Dental problems	1/18 (6%)

Myelomeningocele	1/18 (6%)

## Epidemiology

The incidence of 15q24 deletion syndrome is unknown. To date, there have been 19 published reports of individuals with 15q24 deletion syndrome in which clinical information and detailed mapping of the genomic breakpoints are available [1-10]. The first case was reported in 2007 by Sharp et al [1]. Prior to this time, the widespread use of array CGH was limited, therefore the diagnosis may have gone undetected and undiagnosed. With the advent of array CGH, we anticipate that the present number of reported cases represents an underestimate of the actual prevalence of the disorder. A recent study by Cooper et al [11] reviewed 15,767 cases with intellectual disability and various congenital defects referred to their laboratory for array CGH. They identified 15q24 deletion in nine patients with intellectual disability, for an incidence of approximately 1 in 1751 in this population. This would make it approximately 1/10^th ^as frequent as 22q11.2 deletion syndrome which was identified in 96 cases with intellectual disability (~1 in 164). Given these estimates, and the proposed incidence of 22q11.2 deletion syndrome, the overall incidence of 15q24 microdeletion in the general population may approach approximately 1 in 42,000. We would expect the deletion to occur with equal frequency in males and females; however 16 of the 19 reported cases (84%) are males. The reason for the discrepancy in the male to female sex ratio of 15q24 deletion is not clearly understood and may just be representative of the small sample size of cases and ascertainment bias, rather than a true phenomenon. The deletion occurred as a *de novo *event in the 18 individuals that the parents were tested [1-10].

## Clinical description

The clinical characteristics of 15q24 deletion syndrome are based on the clinical features reported in the 19 patients with 15q24 microdeletion and presented in Table 1[1-10]. At birth, approximately one-third of the individuals had low birth weight consistent with intrauterine growth retardation. Feeding difficulties and failure to thrive were reported in approximately 20%. However, in later childhood, growth parameters were extremely variable with nearly 30% of individuals still exhibiting postnatal growth retardation and short stature, while 17% had obesity. Additionally, almost 20% of individuals were reported to have microcephaly. Two individuals with 15q24 deletion had documented growth hormone (GH) deficiency. This might be an underascertainment of the actual prevalence of GH deficiency in this population since it may not have been assessed in other individuals.

Growth delay, feeding difficulties, and distinct facial features were often the presenting early symptoms of 15q24 deletion syndrome. Distinct facial features, including long face with high anterior hairline, epicanthal folds, hypertelorism, downslanting palpebral fissures, sparse and broad medial eyebrows, broad and/or depressed nasal bridge, long smooth philtrum, and small mouth with full lower lip were common in all individuals. Ear abnormalities were variable, but present in nearly two-thirds of individuals and included large ears, ear lobe pits, anteverted ear lobes, and protuberant ears. These features may be subtle in the newborn period, but may become more evident with age.

All of the individuals have developmental delay of varying degrees, usually in the range of mild to moderate intellectual disability. In reviewing the literature, 6/19 cases were classified as having mild intellectual disability, 2/19 were classified as moderate, 1/19 was classified as moderate-to-severe, 3/19 were classified as severe, and the remaining 7/17 were not further characterized based on severity. Behavior abnormalities, such as autism, hyperactivity, aggression, and attention deficit disorder have been reported in 37% individuals with 15q24 deletion syndrome. McInnes et al performed targeted aCGH across the 15q24 region on ~1500 individuals with autism spectrum disorders and identified a *de novo *atypical deletion of 3.06 Mb in 15q23-q24.1 in a 5 year old male with autism [8]. In addition to autism, he exhibited other characteristic features of 15q24 deletion syndrome including, moderate intellectual disability, low birth weight, distinct facial features, 2,3 toe syndactyly, mild scoliosis, and recurrent respiratory infections. Autism or autistic-like features were reported in three additional individuals with 15q24 deletion [1,9,10].

Skeletal and digital deformities have been described in the majority of individuals. Digit abnormalities such as proximally implanted and/or hypoplastic thumbs, clinodactyly, brachydactyly, overriding toes, toe syndactyly, and small hands were found in approximately 90% of individuals with 15q24 deletion. In addition, many individuals were reported to have joint laxity and scoliosis. Some individuals were reported to have hernias which, in addition to the joint laxity, suggest that there may be an underlying connective tissue abnormality in individuals with 15q24 deletion syndrome [3].

Hypotonia has been reported in 60% of individuals with 15q24 deletion syndrome. Approximately half of the individuals had abnormal brain imaging on magnetic resonance imaging (MRI). The features in those with abnormalities are variable and include: dysplastic or abnormal corpus callosum, cortical atrophy, focal cortical dysplasia, and hypoplastic olfactory bulbs. The incidence of seizure activity is relatively low with only one individual having reported seizures [1].

Genital abnormalities are reported in a majority of individuals (60%) and include hypospadias, microphallus, and cryptorchidism. These findings, in addition to other possible malformations or dysmorphisms in the newborn period, may prompt an earlier genetics evaluation and diagnosis when compared to females who have not been reported to have urogenital anomalies. This may possibly explain why the majority of reported patients are males.

Nearly 40% of individuals with 15q24 deletion syndrome had a history of recurrent infections, such as chest, upper airway, and ear infections. The etiology of the recurrent infections is currently unknown. Immunodeficiency could be the underlying cause; however, immunologic work up has not been reported in any of those individuals. Recurrent ear infections may be one of the factors that predispose to hearing loss which has been formally evaluated and diagnosed in nearly 25% of individuals with 15q24 deletion syndrome.

Birth defects have been reported in several individuals including congenital heart defects (such as tetralogy of Fallot and ventricular septal defects) in four (~20%), intestinal atresia in two, imperforate anus in one [5], iris coloboma in one, myelomeningocele in one, and diaphragmatic hernia in two individuals. Other features that have been reported in a minority of cases include strabismus [1-3,6,10] nystagmus [1,5,8] and dental abnormalities [1].

One individual with 15q24 deletion syndrome was reported to have acute lymphoblastic leukemia [4] and another individual was diagnosed with cardiac rhabdomyoma [7]. No additional malignancies or tumors have been reported in this population, therefore it is currently unknown if there is a predisposition to tumors and neoplasia in individuals with 15q24 deletion syndrome.

Two individuals with 15q24 deletion syndrome had another concurrent chromosome abnormality [7,9]. These individuals exhibited additional clinical features with a more severe phenotype than that observed in previously reported patients with isolated 15q24 rearrangements and it was suggested that the number of genomic rearrangements (genomic mutational load) may contribute to the phenotypic severity and variability in patients with 15q24 deletions [7,12,13].

## Etiology

15q24 deletion syndrome is a microdeletion syndrome resulting from the interstitial loss of 15q24 ranging in size from ~1.7 to 6.1 Mb. All but one case share the smallest region of overlap (SRO) of ~1.2 Mb in size (see Figure 1) [1-10]. The 15q24 deletions are primarily mediated by nonallelic homologous recombination (NAHR) between paralogous low-copy repeats (LCRs). The majority of 15q24 deletions have breakpoints that localize to LCR clusters. Five LCR clusters have been identified, labeled, LCR15q24A, LCR15q24B, LCR15q24C, LCR15q24D, and LCR15q24E. The SRO spans the 1.2 Mb region between LCR15q24B to LCR15q24C. Some patients have had breakpoints that do not lie within the LCR regions. Breakpoint sequencing for the 15q24 deletion in one of those patients revealed microhomology suggesting that other possible mechanisms include nonhomologous end joining (NHEJ) and fork stalling and template switching (FoSTeS)/microhomology-mediate break-induced replication (MMBIR) [7,14-17].

The SRO is a gene-rich region that includes several genes including: *CYP11A1, SEMA7A, CPLX3, ARID3B, STRA6, SIN3A *and *CSK*. *CYP11A1 *encodes the cholesterol side-chain cleavage enzyme and it has been suggested that haploinsufficiency of *CYP11A1 *may contribute to the genital abnormalities in patients with 15q24 deletion syndrome [7]. *SEMA7A *and *CPLX3 *are two genes that may play an important role in brain development and may contribute to the cognitive disability in individuals with this deletion [7]. Haploinsufficiency of *ARID3B *and *STRA6 *may contribute to the congenital malformations observed in a handful of patients with 15q24 deletion syndrome. The *ARID3B *gene is involved with transcription regulation in embryonic development. Homozygous null mutations in the *ARID3B *mouse model predispose to an increased incidence of cardiovascular malformations [18]. Similarly, homozygous loss-of-function mutations in *STRA6 *have also been associated with cardiovascular malformations, microphthalmia, and diaphragmatic hernias [19,20]. *SIN3A *and *CSK *both play roles in tumor suppression and their deletion may lead to an increased predisposition to and risk of neoplasia in this patient population [7].

Apparent reciprocal 15q24 duplications involving the SRO have been reported in 4 individuals [4,7,21]. Two of these individuals shared multiple clinical features with one another, including developmental delay, hypertonia, joint limitations, digital abnormalities, and distinct facial features such as down-slanting palpebral fissures, epicanthal folds, smooth philtrum, and full lower lip [4,21]. However, other reported individuals with the reciprocal 15q24 duplication did not have the same clinical phenotype, therefore, it is unclear whether 15q24 duplications result in a distinct clinical phenotype [7,21].

## Diagnostic methods

Oligonucleotide array CGH with confirmation by fluorescent in-situ hybridization (FISH) detects most, if not all, deletions of 15q24. Routine high-resolution chromosome analysis, when performed, is typically normal. Array CGH will also be able to detect additional chromosome copy number variants (CNVs) that may influence the clinical phenotype. Parental FISH and/or aCGH should be offered to the parents when 15q24 deletion is detected in a child.

## Differential diagnoses

15q24 deletion syndrome should be differentiated from other microdeletion/duplication and genetic syndromes that have similar presentations. Individuals with 22q11.2 deletion syndrome (velo-cardio-facial syndrome) can have distinct facial features, asymmetric facies, and developmental delay. However, congenital heart defects and palate abnormalities are much more common in 22q11.2 deletion syndrome than in 15q24 deletion syndrome. Array CGH or FISH for 22q11.2 deletion will detect this deletion.

There is also significant overlap between the features of 15q24 deletion syndrome and Prader-Willi syndrome (PWS). Prader-Willi syndrome, a genetic condition caused by loss of the paternal copy of 15q11.2, is characterized by neonatal hypotonia, feeding difficulties, and genital abnormalities, such as hypogonadism. In addition, individuals with PWS will typically have mild to moderate intellectual disability, behavior problems, and obesity. The degree of hypotonia and feeding difficulties is more severe in individuals with PWS than what would be expected in newborns with 15q24 deletion syndrome. In addition, obesity in older children with PWS is much more common than what has been reported in individuals with 15q24 deletion. Array CGH, FISH for PWS and/or methylation studies for PWS will detect the majority of cases of Prader-Willi syndrome.

Individuals with Noonan syndrome may share some similarities to individuals with 15q24 deletion syndrome, particularly, distinct facial features, skeletal abnormalities, short stature, cryptorchidsm, failure to thrive, and developmental delay. However the facial features of individuals with Noonan syndrome are more characteristic, whereas the facial features of individuals with 15q24 deletion may be rather subtle, especially in the newborn period. Failure to thrive and feeding difficulties, while common in both conditions, tends to be more severe in Noonan syndrome. Both conditions have a high incidence of skeletal malformations; however pectus deformities are more common in Noonan syndrome, whereas digit abnormalities are predominant in 15q24 deletion syndrome. Molecular sequencing of genes in Ras-MAPK (mitogen-activated protein kinase) pathway will diagnose Noonan syndrome (and related conditions) in the majority of cases.

## Genetic counseling

Variable expressivity exists between individuals with 15q24 deletion syndrome. Some of the variability may be attributed to the specific size and location of the deletion, however, even individuals with similar breakpoints exhibit diverse phenotypes. Offering accurate anticipatory guidance and counseling for individuals with 15q24 deletion syndrome can be challenging especially if the individual has additional genomic rearrangements that may predispose to other clinical consequences. This may interfere with the clinicians' ability to provide an accurate assessment or outcome in the child. The phenotype, clinical features, and prognosis will depend on the size, location, and genes involved in the additional chromosome abnormality.

The combination of the additional rearrangements with the 15q24 deletion may have an additive effect as the presence of multiple genomic rearrangement in an individual (the genomic mutational load) can result in more severe phenotype and additional features that are not typically observed in individuals carrying single rearrangements [7,12,13,22]. Genetic counseling should take into account this potential effect and be attentive to additional phenotypic variability that may occur.

To date, all of the cases where parents were available for testing (18 out of 19 cases) indicated that the deletion occurred as a *de novo *event in the child. However, for genetic counseling, reproductive planning, and anticipatory guidance, it is recommended that both parents have FISH or aCGH testing in order to confirm that neither parent also carries the deletion. This is particularly important if the individual has an additional chromosome abnormality or imbalance that may have been inherited from a balanced rearrangement in one of the parents.

Individuals with 15q24 deletion syndrome have a 50% chance of passing on the deletion to their offspring. To date, although the two oldest reported individuals are 33 years old, there have not been any reported cases of individuals with 15q24 deletion syndrome reproducing, therefore, it is unknown if there are any fertility-related issues in this population. Longitudinal follow-up of adolescence and adults with 15q24 deletion will help determine if the high incidence of genital abnormalities predisposes to fertility issues later in life.

## Antenatal diagnosis

Deletion of 15q24 can be detected in amniotic fluid or chorionic villi samples. Since routine karyotyping is not typically sufficient to detect the deletion, aCGH should be offered to parents who carry a structural rearrangement that may predispose to 15q24 deletion or if fetal abnormalities are noted antenatally. Prenatal ultrasonography may detect some cardiovascular malformations, diaphragmatic hernias, and growth retardation in the fetus with 15q24 deletion, however, the most characteristic features of the condition, specifically distinct facial features and digit abnormalities, may be difficult to detect.

## Management

The proposed management recommendations are based on the current knowledge of the small number of patients reported with this condition in conjunction with the authors' clinical experience, and are meant to serve as a guideline for treating individuals with this condition.

Routine medical management of individuals with 15q24 deletion syndrome and coordination of referrals can be performed by the individual's primary care physician, however most individuals will require multidisciplinary care from a variety of subspecialists (Table 2). Initial presenting features may include failure to thrive, feeding difficulties, hypotonia, genital anomalies, and distinct facial features. Referral to a clinical geneticist is indicated in any child with a combination of these findings. Detailed physical examination, medical history, and developmental assessment should prompt genetic testing such as aCGH, which will diagnose microdeletions of 15q24. Periodic evaluation by a clinical geneticist is warranted to update the family on new information or symptoms that may be related to 15q24 deletion syndrome.

**Table 2:** Recommended clinical evaluations and management for individuals with 15q24 deletion syndrome

*Specialist*	*Evaluation at time of diagnosis*	*Screening and surveillance*
Primary care physician	Initiate referrals to subspecialist, monitor growth and development	Assess growth parameters and development at each visit

Development clinic	Neurocognitive (developmental) and behavioral evaluation	At time of diagnosis, then annually in childhood

Geneticist	Confirmation of diagnosis and periodic follow-up evaluations	At time of diagnosis, then annually

Audiology	Routine audiology evaluation	At time of diagnosis, then as needed.

Ophthalmology	Evaluate for coloboma, visual acuity, nystagmus, strabismus	At time of diagnosis, then as needed.

Cardiology	Echocardiogram to evaluate for congenital heart defects	At time of diagnosis, then as needed

Allergy and Immunology	Immune work-up for recurrent and persistent infections	As needed

Endocrine	Growth hormone studies for growth retardation	As needed

Gastroenterology/Feeding and Nutrition	Feeding assessment for failure to thrive and growth retardation	As needed

Neurology	Brain MRI for structural malformations and EEG for seizures	As needed

Orthopedics	Spine x-rays and scoliosis evaluation	As needed

Urology	Evaluate for genital abnormalities	As needed

### Growth and Feeding

The primary care physician should closely monitor all growth parameters in any individual diagnosed with 15q24 deletion syndrome. Referral to a gastroenterologist and/or feeding and nutrition clinic is warranted in any infant with 15q24 deletion syndrome and feeding difficulties, failure to thrive, or improper weight gain. Because of the risk of childhood obesity in individuals with 15q24 deletion syndrome, weight should be closely monitored even in those subjects with normal growth parameters. Nutritional counseling is recommended in those individuals who experience excessive weight gain. For children with growth retardation, evaluation by an endocrinologist for GH stimulation studies is also recommended. Although no data is available about the effect of GH treatment in individuals with 15q24 deletion, we would except that such treatment would be helpful especially in those with documented GH deficiency.

### Development and Behavior

Given the high incidence of developmental delay, intellectual disability, and behavior problems in children with 15q24 deletion syndrome, formal neurocognitive and behavior assessment is recommended to determine the child's specific learning pattern and behavioral profile. Behavioral therapy may benefit individuals with this syndrome. Therefore periodic follow-up with a developmental and/or behavioral specialist is warranted. Early childhood intervention, encompassing physical therapy, occupational therapy, and speech therapy is needed in most of the cases.

### Neurologic manifestations

As structural brain anomalies have been reported in approximately half of all of cases of 15q24 deletion, brain magnetic resonance imaging (MRI) studies are indicated in any individual with 15q24 deletion and abnormal neurological examination. Referral to neurology is needed in individuals with structural brain malformations or if seizures are suspected.

### Cardiac manifestations

A baseline echocardiogram is recommended in any individual with the new diagnosis of 15q24 deletion syndrome. Follow-up cardiology evaluations and echocardiograms should be performed based on the results of initial echocardiogram and at the discretion of the patient's cardiologist.

### Ophthalmologic manifestation

Ophthalmologic abnormalities are common in individuals with 15q24 deletion syndrome; therefore, baseline ophthalmologic evaluations are recommended to screen for structural eye abnormalities and periodic ophthalmologic evaluations should be performed to screen for nystagmus and strabismus.

### Orthopedic manifestations

Physical examination of the curvature of the spine needs to be evaluated with every routine care visit. Spine x-rays are indicated if the child shows any signs of scoliosis and/or kyphosis. If scoliosis and/or kyphosis develop then referral to orthopedics is warranted for follow up and discussing various treatment options.

### Urogenital abnormalities

A majority of the males with 15q24 have genital abnormalities. Referral to urology for evaluation of the genital abnormalities, if present, is recommended in order to determine if surgical correction or any other treatment is indicated.

### Recurrent infections

Individuals with 15q24 deletion syndrome are at risk for recurrent chest, upper airway, and ear infections. To date, documented immune deficiency has not been reported in any individual with 15q24 deletion syndrome, however, a referral to immunology may be warranted for recurrent and persistent infections. Referral to otolaryngology is recommended in case of recurrent otitis media and a low threshold is needed for placing pressure equalization (PE) tubes in children with 15q24 deletion syndrome. Recurrent otitis media may also lead to conductive hearing loss, which may affect a child's expressive and receptive language development; therefore, periodic audiologic evaluation is also indicated.

## Prognosis

The prognosis for individuals with 15q24 deletion syndrome depends on the severity and extent of congenital malformations. Major birth defects have been reported including, tetralogy of Fallot, myelomeningocele, intestinal atresia, and congenital diaphragmatic hernia (CDH). The prognosis for any individual with CDH is guarded and related to size, location, and visceral involvement of the diaphragmatic hernia [23]. In general, the majority of individuals with 15q24 deletion typically do not have life-threatening organ malformations. The natural history of 15q24 deletion syndrome is currently unknown since there are very few children and even fewer adolescents and adults reported with this deletion. The oldest reported patients, who were 33-years-old at the time of their report, have variable outcome [1,3]. The individual reported by Sharp et al. is a 33-year-old male who had mild developmental delay as a child. He has short stature and relatively mild intellectual disability with good language skills as an adult [1]. Van Esch et al. also reported a 33-year-old male who had severe developmental delay and intellectual disability, hypotonia, and limited speech [3]. He had hyperactive behavior with aggressive outbursts in childhood and adolescence and remained difficult to handle in adulthood despite psychopharmacological therapy. Longitudinal follow-up of individuals with this deletion will clarify the developmental and functional level of independence that individuals may expect to have in adulthood and possible health-related complications as individuals with this deletion age.

## Unresolved questions

Since 15q24 deletion syndrome is a relatively newly described condition there are still many questions regarding the clinical manifestations that remain unanswered. Salient features that have been reported in a minority of individuals, but have the potential to drastically affect prognosis and anticipatory guidance of individuals with this condition, are the occurrence of neoplasias. At this time, it is unknown if there is a causal relationship between 15q24 deletion and the neoplasias described. Therefore, clinicians should be cognizant of the possible risk of tumors in this population and have low threshold for evaluation if neoplasia is remotely suspected.

The underlying explanation for the discrepancy in the male to female ratio of reported individuals remains unknown. As previously mentioned, this may represent an ascertainment bias rather than a true disparity. A more balanced ratio would be expected as more individuals with 15q24 deletion syndrome are identified.

Lastly, the clinical and phenotypic consequence of the reciprocal duplication of 15q24 remains unknown. Identification of more individuals with 15q24 duplication will enable better classification of clinical features that may be associated with the duplication and a better understanding of the molecular mechanisms that precipitate both the 15q24 deletion and reciprocal duplication.

## Future prospects

Further characterization of the clinical phenotype, genotype-phenotype correlations, medical complications, and developmental potential of individuals with 15q24 deletion syndrome is imperative in order to fully comprehend the variability and severity of the syndrome. Natural history studies and longitudinal follow-up by the clinician will provide beneficial information on how children, adolescents, and adults with 15q24 deletion age and progress over time. This could lead to the development of practical screening, management, and surveillance guidelines for any individual diagnosed with 15q24 deletion syndrome.

## List of abbreviations

aCGH: array comparative genomic hybridization; NAHR: non-allelic homologous recombination; LCR: low-copy repeats; SRO: smallest region of overlap; MRI: magnetic resonance imaging; GH: growth hormone; NHEJ: nonhomologous end joining; FoSTeS: fork stalling and template switching; MMBIR: microhomology-mediate break-induced replication; FISH: fluorescent in-situ hybridization; CNV: copy number variant; PWS: Prader-Willi syndrome; MAPK: mitogen-activated protein kinase; EEG: electroencephalogram; PE: pressure equalization; CDH: congenital diaphragmatic hernia.

## Competing interests

The authors declare that they have no competing interests.

## Authors' contributions

Both authors participated in writing this review. They read and approved the final version of the manuscript.
